# Genetic heterogeneity and transcriptional plasticity drive resistance during immunotherapy and targeted therapy

**DOI:** 10.1186/1479-5876-13-S1-K18

**Published:** 2015-01-15

**Authors:** Reinard Dummer

**Affiliations:** 1 University of Zurich Hospital, Department of Dermatology, Zurich, Switzerland

## 

Cancer is caused by genetic, epigenetic and microenvironmental changes that facilitate the survival and proliferation of tumor cells and their ability to acquire invasive properties. The plasticity of human tumor cells generally replicates normal molecular processes occurring during development and tissue repair. In humans, cancer progression is also shaped by host immune responses that edit the final tumor-host interactions. The genetic complexity and extreme variability of human melanoma means a multidisciplinary integrative approach is needed to understand the interactions between the genetic background of the host, the tumor and its microenvironment, and the impact of these on the immune system. It is becoming evident that successful anti-tumor strategies need to encompass a multimodal approach to avoid tumor escape or relapse, combining agents able to block essential signal transduction pathways with immunotherapy.

